# Supplementary material for: Targeted Analysis of the Gut Microbiome for Diagnosis, Prognosis and Treatment Individualization in Pediatric Inflammatory Bowel Disease
Source: Microorganisms. 2022 Jun 22;10(7):1273. doi: 10.3390/microorganisms10071273 (PMC9319120; doi:10.3390/microorganisms10071273)
Supplement: Supplementary file 1 [file microorganisms-10-01273-s001.zip › Supplementary Table S1.pdf]

Supplementary Table S1. Basic PMP™ panels

**PMP1<br>BIOMEPMP-001 ver 1**	**PMP2<br>BIOMEPMP-002 ver 1**
Akkermansia muciniphila	*Acidaminococcus intestini*
Alistipes finegoldii	*Alistipes onderdonkii*
Alistipes putredinis	*Alistipes shahii*
Anaerobutyricum hallii	*Anaerostipes hadrus*
Bacteroides caccae	*Bacteroides coprocola*
Bacteroides cellulosilyticus	*Bacteroides fragilis*
Bacteroides dorei	*Bacteroides intestinalis*
Bacteroides eggerthii	*Bacteroides nordii*
Bacteroides finegoldii	*Bacteroides plebeius*
Bacteroides massiliensis	*Bacteroides pyogenes*
Bacteroides ovatus	*Bacteroides stercoris*
Bacteroides stercorirosoris	*Bacteroides uniformis*
Bacteroides thetaiotaomicron	*Bifidobacterium breve*
Bacteroides vulgatus	*Bilophila wadsworthia*
Bacteroides xylanisolvens	*Butyrivibrio crossotus*
Barnesiella intestinihominis	*Citrobacter koseri*
Bifidobacterium adolescentis	*Clostridium bolteae*
Bifidobacterium angulatum	*Clostridium butyricum*
Bifidobacterium animalis lactis	*Clostridium citroniae*
Bifidobacterium bifidum	*Clostridium nexile*
Bifidobacterium catenulatum	*Clostridium perfringens*
Bifidobacterium longum	*Clostridium sporogenes*
Bifidobacterium longum infantis	*Clostridium symbiosum*
Bifidobacterium longum longum	*Collinsella intestinalis*
Bifidobacterium pseudocatenulatum	*Coprococcus catus*
Blautia hydrogenotrophica	*Desulfovibrio piger*
Christensenella minuta	*Eggerthella lenta*
Clostridium leptum	*Enterococcus dispar*
Clostridium scindens	*Enterococcus faecalis*
Collinsella aerofaciens	*Enterococcus faecium*
Coprococcus comes	*Enterococcus hirae*
Dorea formicigenerans	*Eubacterium siraeum*
Dorea longicatena	*Fusobacterium varium*
Erysipelatoclostridium ramosum	*Haemophilus parainfluenzae*
Escherichia coli	*Hafnia alvei*

Eubacterium eligens	*Holdemanella biformis*
Eubacterium rectale	*Lactobacillus animalis*
Eubacterium ventriosum	*Lactobacillus brevis*
Faecalibacterium prausnitzii	*Lactobacillus reuteri*
Klebsiella variicola	*Morganella morganii*
Lactobacillus acidophilus	*Mycoplasma hominis*
Lactobacillus paracasei	*Odoribacter splanchnicus*
Lactobacillus ruminis	*Parabacteroides goldsteinii*
Methanobrevibacter smithii	*Parabacteroides gordonii*
Parabacteroides distasonis	*Paraprevotella clara*
Parabacteroides merdae	*Prevotella stercorea*
Prevotella copri	*Proteus mirabilis*
Roseburia hominis	*Roseburia inulinivorans*
Roseburia intestinalis	*Ruminococcus albus*
Ruminococcus gnavus	*Ruminococcus bromii*
Streptococcus thermophilus	*Ruminococcus torques*
Subdoligranulum variabile	*Streptococcus sanguinis*
Sutterella wadsworthensis	*Turicibacter sanguinis*
Homo sapiens*	*Veillonella atypica*
Prokaryotic 16S rRNA gene target*	Prokaryotic 16S rRNA gene target*
Xeno IPC*	Xeno IPC*

* Control assays
