# Supplementary material for: Targeted Analysis of the Gut Microbiome for Diagnosis, Prognosis and Treatment Individualization in Pediatric Inflammatory Bowel Disease
Source: Microorganisms. 2022 Jun 22;10(7):1273. doi: 10.3390/microorganisms10071273 (PMC9319120; doi:10.3390/microorganisms10071273)
Supplement: Supplementary file 1 [file microorganisms-10-01273-s001.zip › Supplementary Table S2 .pdf]

Supplementary Table S2:
The species targeted by PM^{PTM} panels. The PMP^{TM} qPCR probes are specific for the commercially available DMSZ and ATCC bacteria species genomes listed, and standard curves are made for each reference genome to enable quantification of the bacteria species.

For independent validation by external and independent investigators, the validated qPCR assays can be ordered from ThermoFisher Scientific (https://thermofisher.com/, catalogue number 4471120 Custom OpenArray and referring to specific Custom OpenArray ID name provided.

ThermoFisher panel name: BIOMEPMP-001 ver 1			
Target name	Standard curve coefficients		Reference material
	Slope	Intercept	
Akkermansia muciniphila	-0,992000687	27,99878359	DSMZ 22959<br>DSMZ 26127
Alistipes finegoldii	-0,987015592	30,05656981	DSMZ 17242
Alistipes putredinis	-1,09219549	31,50681812	DSMZ 17216
Anaerobutyricum hallii	-0,944502753	30,52602934	DSMZ 3353
Bacteroides caccae	-0,94871781	29,78049382	DSMZ 19024
Bacteroides cellulosilyticus	-1,015547033	30,26067387	DSMZ 14838
Bacteroides eggerthii	-0,978390031	28,12306779	DSMZ 20697
Bacteroides finegoldii	-0,958395567	29,34717734	DSMZ 17565
Bacteroides ovatus	-1,029435869	30,55452496	DSMZ 1896
Bacteroides stercorirosoris	-0,969056961	28,56165362	DSMZ 26884
Bacteroides thetaiotaomicron	-0,995885261	29,97868842	DSMZ 2079
Bacteroides xylanisolvens	-1,049771232	30,2109513	DSMZ 18836
Barnesiella intestinihominis	-0,994232393	29,2892562	DSMZ 21032
Bifidobacterium adolescentis	-1,016812826	27,59507437	DSMZ 20083
Bifidobacterium angulatum	-0,97819653	29,71230634	DSMZ 20098
Bifidobacterium animalis subsp. lactis	-0,962323335	27,04370951	DSMZ 10140
Bifidobacterium bifidum	-0,965109065	30,14128241	DSMZ 20456
Bifidobacterium catenulatum	-0,960286326	28,05646253	DSMZ 16992
Bifidobacterium longum	-0,972813445	28,90702869	DSMZ 20219<br>DSMZ 20088
Bifidobacterium longum subsp. infantis	-0,974530288	30,31297195	DSMZ 20088
Bifidobacterium longum subsp. longum	-1,002661994	30,54039514	DSMZ 20219
Bifidobacterium pseudocatenulatum	-0,991734883	26,5329584	DSMZ 20438
Blautia hydrogenotrophica	-1,009713351	30,8131316	DSMZ 10507
Christensenella minuta	-0,915931057	26,26144741	DSMZ 22607
Clostridium leptum	-0,974275484	26,94196736	DSMZ 753
Clostridium scindens	-0,992494637	27,84473891	DSMZ 5676
Collinsella aerofaciens	-0,995470113	29,80091144	DSMZ 3979
Coprococcus comes	-0,973421585	28,83727187	ATCC 27758
Dorea formicigenerans	-0,99322225	29,6728366	DSMZ 3992
Dorea longicatena	-1,001829167	30,09620124	DSMZ 13814
Erysipelatoclostridium ramosum	-1,033754514	30,21368043	DSMZ 1402
Escherichia coli	-0,973574125	29,26532652	DSMZ 30083
Eubacterium eligens	-0,981951481	30,41186476	DSMZ 3376
Eubacterium rectale	-0,99768899	27,90880377	DSMZ 17629
Eubacterium ventriosum	-0,949532018	29,78624085	DSMZ 3988
Faecalibacterium prausnitzii	-0,99727617	30,26448204	DSMZ 17677
Klebsiella variicola	-0,967341238	29,58339885	DSMZ 15968
Lactobacillus acidophilus	-1,017483886	30,54203154	DSMZ 20079
Lactobacillus paracasei	-1,02335	31,48621617	DSMZ 5622
Lactobacillus ruminis	-0,982129925	29,68251303	DSMZ 20403
Methanobrevibacter smithii	-1,027102828	29,23576913	DSMZ 861
Parabacteroides distasonis	-0,980092589	28,75145783	DSMZ 20701
Parabacteroides merdae	-0,99997555	29,050045	DSMZ 19495
Phocaeicola dorei	-0,915311	28,32832543	DSMZ 17855
Phocaeicola massiliensis	-0,980048365	28,272182	DSMZ 17679
Phocaeicola vulgatus	-0,993751661	30,04611268	DSMZ 1447
Prevotella copri	-0,981407637	28,8991566	DSMZ 18205<br>DSMZ 108494
Roseburia hominis	-1,022234339	30,31886566	DSMZ 16839
Roseburia intestinalis	-1,041619788	27,450061	DSMZ 14610
Ruminococcus gnavus	-1,003818109	29,66311384	DSMZ 108212
Streptococcus thermophilus	-0,976811059	29,89745222	DSMZ 20617
Subdoligranulum variabile	-0,989364836	29,29031483	DSMZ 15176
Sutterella wadsworthensis	-0,964743202	28,73388786	DSMZ 14016

ThermoFisher panel name: BIOMEPMP-002 ver 1			
**Target name**	**Standard curve coefficients**		**Reference material**
	**Slope**	**Intercept**	
Acidaminococcus intestini	-1,050610476	30,97420279	DSMZ 21505
Alistipes onderdonkii	-1,04163499	30,7075846	DSMZ 19147
Alistipes shahii	-1,059184212	30,26379342	DSMZ 19121
Anaerostipes hadrus	-0,992907804	30,08062728	DSMZ 3319
Bacteroides coprocola	-1,03990522	30,12786534	DSMZ 17136
Bacteroides fragilis	-1,048859137	30,02264856	DSMZ 2151
Bacteroides intestinalis	-1,016988643	29,74175488	DSMZ 17393
Bacteroides nordii	-1,099235729	30,93153838	DSMZ 18764
Bacteroides plebeius	-1,058616603	30,28596137	DSMZ 17135
Bacteroides pyogenes	-1,271816027	33,48445974	DSMZ 20611
Bacteroides stercoris	-1,044361951	30,52559276	DSMZ 19555
Bacteroides uniformis	-0,980966626	29,21102888	DSMZ 6597
Bifidobacterium breve	-0,980966626	29,21102888	DSMZ 20213
Bilophila wadsworthia	-1,011174288	29,55859266	DSMZ 11045
Butyrivibrio crossotus	-1,039712657	31,01461527	DSMZ 2876
Citrobacter koseri	-1,101893984	31,33504815	DSMZ 4595
Clostridium bolteae	-1,000740792	29,47113956	DSMZ 15670
Clostridium butyricum	-1,063254937	30,86244565	DSMZ 10702
Clostridium citroniae	-1,038895552	30,32929622	DSMZ 19261
Clostridium nexile	-1,072687772	30,80280579	DSMZ 1787
Clostridium perfringens	-0,980966626	29,21102888	DSMZ 756
Clostridium sporogenes	-1,029310664	29,46253709	DSMZ 795
Clostridium symbiosum	-1,042604195	30,29530036	DSMZ 934
Collinsella intestinalis	-1,093034185	30,50259202	DSMZ 13280
Coprococcus catus	-1,087747257	31,09799167	ATCC 27761
Desulfovibrio piger	-1,069730848	30,06719604	DSMZ 749
Eggerthella lenta	-1,066051375	30,99166682	DSMZ 2243
Enterococcus dispar	-1,070386023	30,14395012	DSMZ 6630
Enterococcus faecalis	-1,132995928	31,09974288	DSMZ 20478
Enterococcus faecium	-1,060614174	30,69427997	DSMZ 20477
Enterococcus hirae	-1,082607052	31,15199318	DSMZ 20160
Eubacterium siraeum	-0,990460257	29,86540549	DSMZ 15702
Fusobacterium varium	-1,026206679	31,05126535	DSMZ 19868
Haemophilus parainfluenzae	-1,053668256	29,73433083	DSMZ 8978
Hafnia alvei	-1,069053125	30,0326339	DSMZ 30163
Holdemanella biformis	-1,017959258	27,49219221	DSMZ 3989
Lactobacillus animalis	-1,064022981	30,18071147	DSMZ 20602
Lactobacillus brevis	-1,07579118	31,07036533	DSMZ 20054
Lactobacillus reuteri	-1,0427	29,9435	DSM 20016
Morganella morganii	-1,067576165	29,66653129	DSMZ 30164
Mycoplasma hominis	-1,053821719	31,24076181	DSMZ 25592
Odoribacter splanchnicus	-1,06846044	30,45616819	DSMZ 20712
Parabacteroides goldsteinii	-1,057144887	30,39481878	DSMZ 19448
Parabacteroides gordonii	-0,980966626	29,21102888	DSMZ 19448
Paraprevotella clara	-1,037033793	30,52026965	DSMZ 19731
Prevotella stercorea	-1,12538489	31,84220512	DSMZ 18206
Proteus mirabilis	-1,056509523	31,0140791	DSMZ 4479
Roseburia inulinivorans	-1,058049353	30,1451495	DSMZ 16841
Ruminococcus albus	-1,142339629	30,23344	DSMZ 20455
Ruminococcus bromii	-1,003167843	29,49455476	ATCC 27255
Ruminococcus torques	-1,05778764	31,54960136	ATCC 27756
Streptococcus sanguinis	-1,028696857	28,80567265	DSMZ 20567
Turicibacter sanguinis	-0,986753267	30,71085981	DSMZ 14220
Veillonella atypica	-1,028312462	30,39838561	DSMZ 20739

ThermoFisher panel name: OA56_order OA12			
**Target name**	**Standard curve coefficients**		**Reference material**
	**Slope**	**Intercept**	
Faecalibacterium prausnitzii A2-165	-1,091261592	28,02684149	DSMZ 17677
Faecalibacterium prausnitzii L2-6	-0,969009792	30,42970389	DSMZ 107839
Flavonifractor plautii	-1,058744535	29,18513455	DSMZ 6740
Gordonibacter pamelaeae	-1,12512542	29,08707595	DSMZ 19378
Parvimonas micra	-0,944072827	27,99816219	DSMZ 20468
Peptostreptococcus anaerobius	-1,098427708	30,51909611	DSMZ 2949
Porphyromonas uenonis	-1,220508279	33,52904619	DSMZ 23387

ThermoFisher panel name: OA-13 OA56			
**Target name**	**Standard curve coefficients**		**Reference material**
	**Slope**	**Intercept**	
Bacteroides faecis	-1,024040874	29,7350675	DSMZ 24798
Dialister pneumosintes	-1,027384113	30,55101427	DSMZ 11619
Granulicatella adiacens	-0,972735315	28,66633644	DSMZ 9848
Hungatella hathewayi	-1,005325141	29,59918054	DSMZ 13479
Parasutterella excrementihominis	-1,055130127	28,27263925	DSMZ 21040
Phascolarctobacterium faecium	-0,989381654	29,38531919	DSMZ 14760
Porphyromonas asaccharolytica	-1,019387495	29,84657111	DSMZ 20707

ThermoFisher panel name: OA 14_OA56			
**Target name**	**Standard curve coefficients**		**Reference material**
	**Slope**	**Intercept**	
Akkermansia muciniphila AmI	-0,994845571	28,44273006	DSMZ 22959, DSMZ 26127
Alistipes communis	-1,122356667	30,25728777	DSMZ 108979
Alistipes senegalensis	-0,979281556	30,02548711	DSMZ 25460
Anaerococcus vaginalis	-1,032004099	29,46349846	DSMZ 7457
Anaerotruncus colihominis	-1,002021992	30,24854647	DSM 17241
Bacteroides salyersiae	-1,025262313	29,54060126	DSMZ 18765
Blautia wexlerae	-0,995536796	29,64784611	DSMZ 19850
Clostridium spiroforme	-0,972878333	28,996124	DSMZ 1552
Dialister invisus	-1,038967067	29,26946106	DSMZ 15470
Fusobacterium nucleatum subsp. animalis	-0,827927655	28,00772805	DSMZ 19679
Fusobacterium nucleatum subsp. nucleatum	-0,978895669	27,21540068	DSMZ 15643
Gemmiger formicilis	-1,034832873	30,11870485	*
Holdemania filiformis	-1,00281002	30,81153709	DSMZ 12042
Intestinibacter bartlettii	-1,007972504	29,75053843	DSMZ 16795

* No target available, Standard curve coefficients determined based on averages
