# Supplementary figures and images for: Targeted Analysis of the Gut Microbiome for Diagnosis, Prognosis and Treatment Individualization in Pediatric Inflammatory Bowel Disease
Source: Microorganisms. 2022 Jun 22;10(7):1273. doi: 10.3390/microorganisms10071273 (PMC9319120; doi:10.3390/microorganisms10071273)

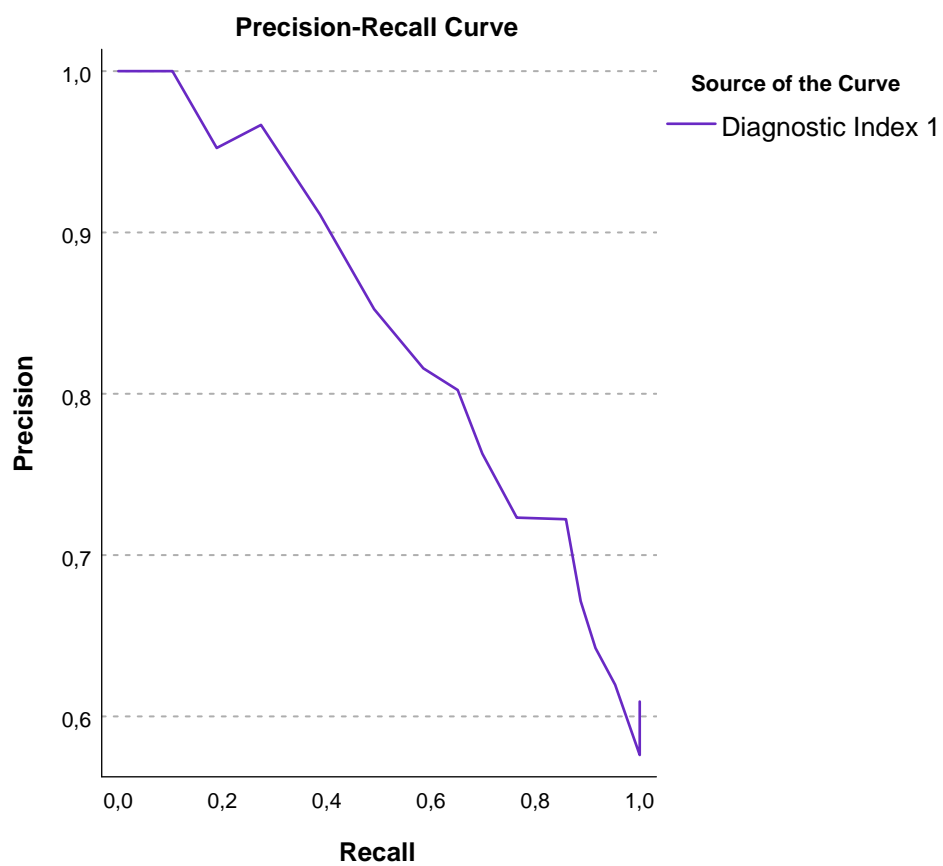

Supplement: Supplementary file 1 [file microorganisms-10-01273-s001.zip › Figure S1_suppl_AUPRC Diagnostic Index 1.pdf]

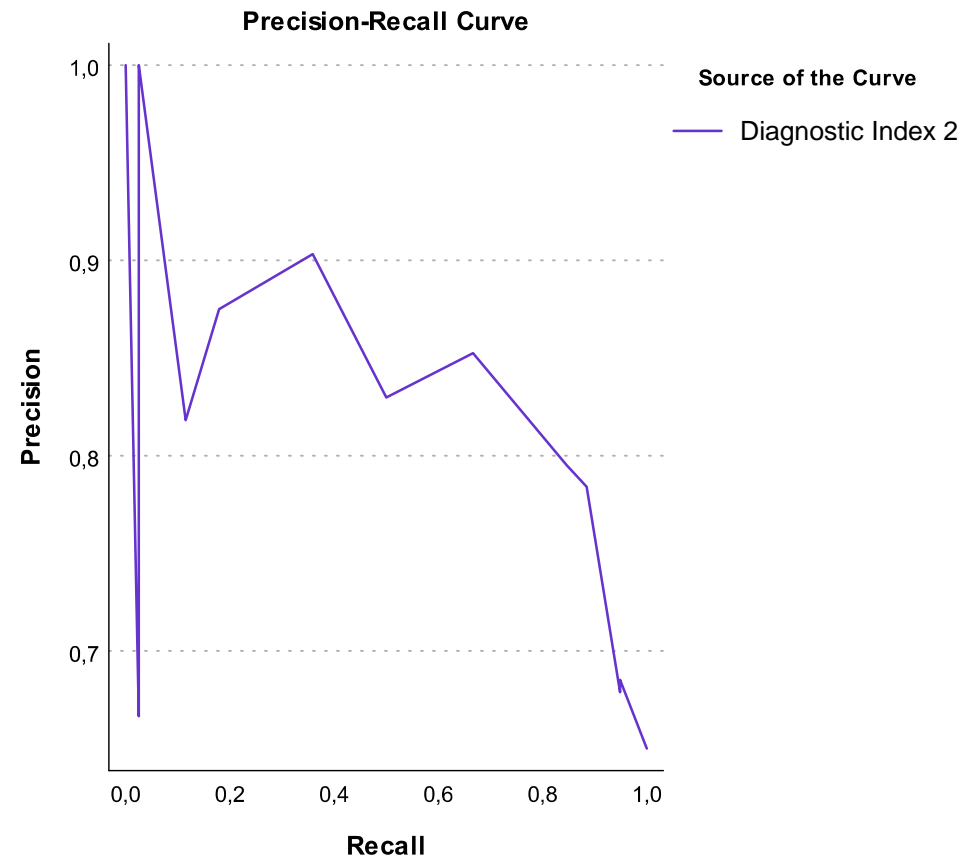

Supplement: Supplementary file 1 [file microorganisms-10-01273-s001.zip › Figure S2_suppl_AUPRC Diagnostic Index 2.pdf]

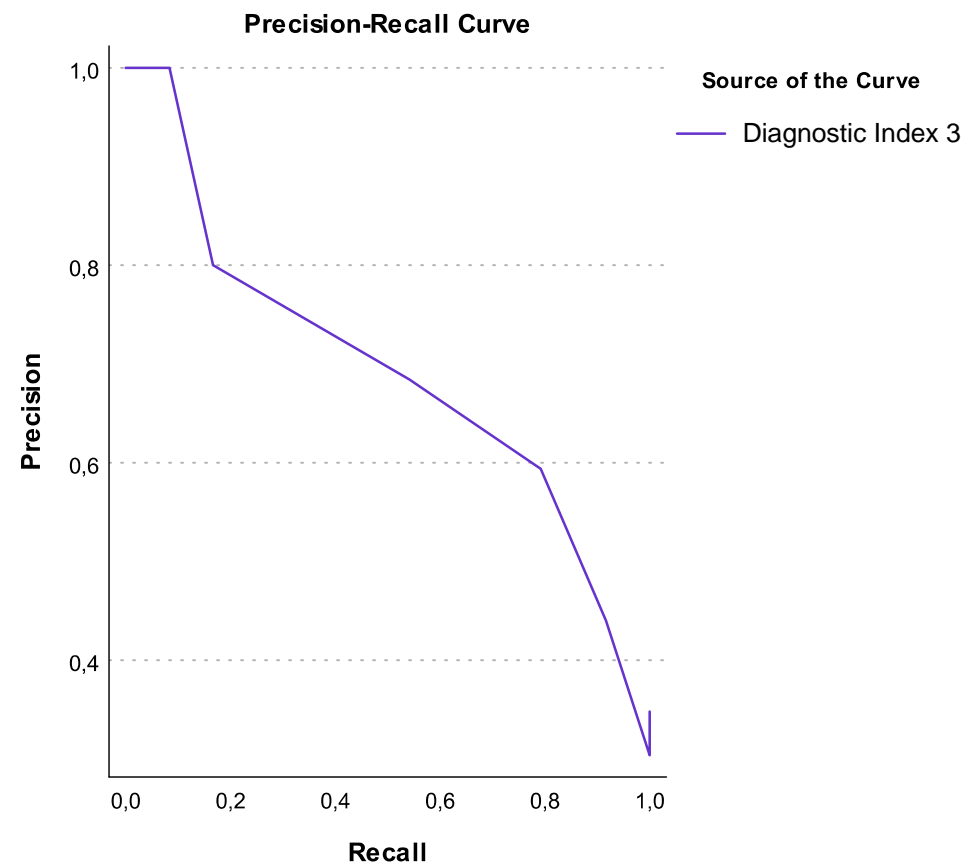

Supplement: Supplementary file 1 [file microorganisms-10-01273-s001.zip › Figure S3_suppl_AUPRC Diagnostic Index 3.pdf]

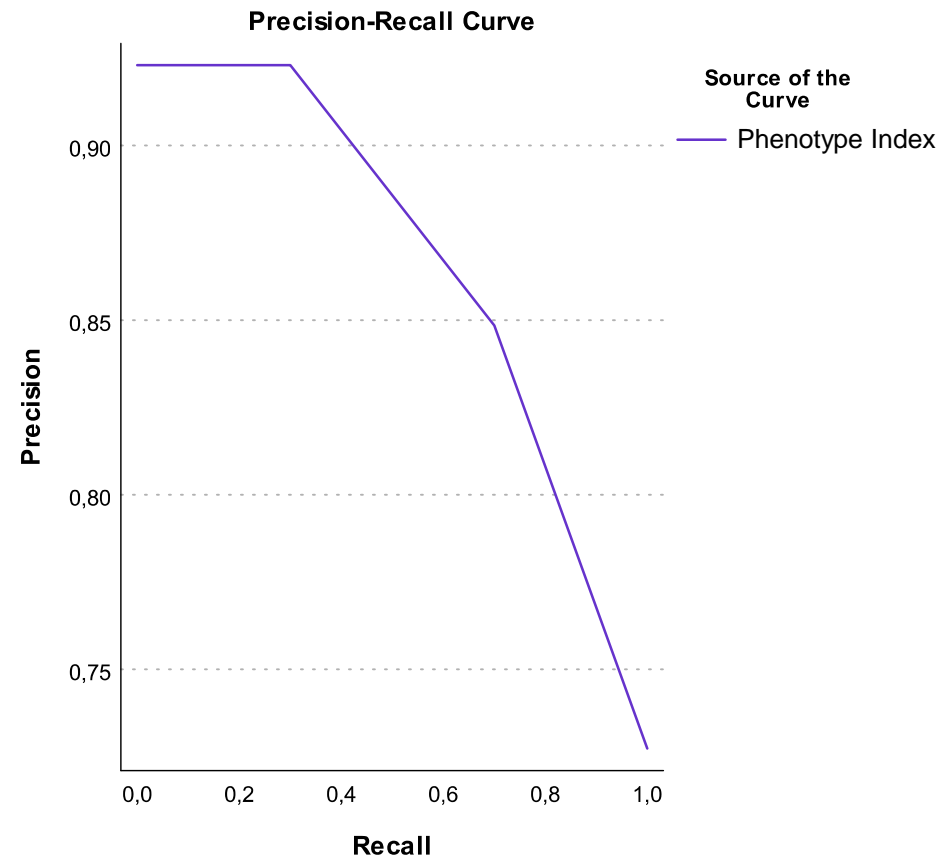

Supplement: Supplementary file 1 [file microorganisms-10-01273-s001.zip › Figure S4_suppl_AUPRC Phenotype Index.pdf]

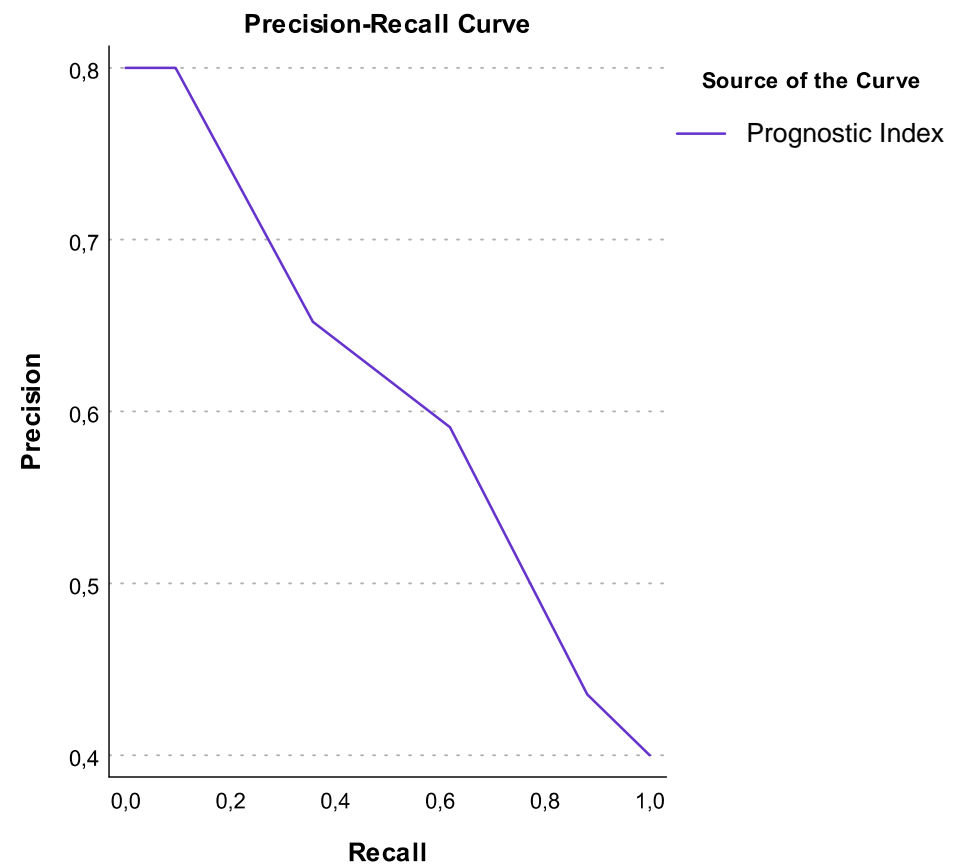

Supplement: Supplementary file 1 [file microorganisms-10-01273-s001.zip › Figure S5_suppl_AUPRC Prognostic Index.pdf]
